# A Metabolome-Wide Study of Dry Eye Disease Reveals Serum Androgens as Biomarkers

**DOI:** 10.1016/j.ophtha.2016.12.011

**Published:** 2017-04

**Authors:** Jelle Vehof, Pirro G. Hysi, Christopher J. Hammond

**Affiliations:** 1 Department of Twin Research & Genetic Epidemiology, King's College London, St. Thomas' Hospital, London, United Kingdom; 2 Department of Ophthalmology, King's College London, St. Thomas' Hospital, London, United Kingdom; 3 Department of Ophthalmology, University of Groningen, University Medical Center Groningen, Groningen, The Netherlands; 4 Department of Epidemiology, University of Groningen, University Medical Center Groningen, Groningen, The Netherlands

**Keywords:** BMI, body mass index, DED, dry eye disease, DHEA, dehydroepiandrosterone, DHEA-S, dehydroepiandrosterone sulfate, SD, standard deviation, SQDES, Short Questionnaire for Dry Eye Syndrome

## Abstract

**Purpose:**

To test the association between serum metabolites and dry eye disease (DED) using a hypothesis-free metabolomics approach.

**Design:**

Cross-sectional association study.

**Participants:**

A total of 2819 subjects from the population-representative TwinsUK cohort in the United Kingdom, with a mean age of 57 years (range, 17–82 years).

**Methods:**

We tested associations between 222 known serum metabolites and DED. All subjects underwent nontargeted metabolomic analysis of plasma samples using gas and liquid chromatography in combination with mass spectrometry (Metabolon Inc., Durham, NC). Dry eye disease was defined from the validated Short Questionnaire for Dry Eye Syndrome (SQDES) as a previous diagnosis of DED by a clinician or “often” or “constant” symptoms of dryness and irritation. Analyses were performed with linear mixed effect models that included age, BMI, and sex as covariates, corrected for multiple testing.

**Main Outcome Measures:**

Primary outcome was DED as defined by the SQDES, and secondary outcomes were symptom score of DED and a clinical diagnosis of DED.

**Results:**

Prevalence of DED as defined by the SQDES was 15.5% (n = 436). A strong and metabolome-wide significant association with DED was found with decreased levels of the metabolites androsterone sulfate (*P* = 0.00030) and epiandrosterone sulfate (*P* = 0.00036). Three other metabolites involved in androgen metabolism, 4-androsten-3beta,17beta-diol disulfate 1 and 2, and dehydroepiandrosterone sulfate, were the next most strongly associated of the 222 metabolites, but did not reach metabolome-wide significance. Dryness and irritation symptoms, as opposed to a clinical diagnosis, were particularly strongly associated with decreased androgen steroid metabolites, with all reaching metabolome-wide significance (androsterone sulfate, *P* = 0.000000029; epiandrosterone sulfate, *P* = 0.0000040; 4-androsten-3beta,17beta-diol disulfate 1, *P* = 0.000016; 4-androsten-3beta,17beta-diol disulfate 2, *P* = 0.000064; and dehydroepiandrosterone sulfate, *P* = 0.00011). Of these 5 androgens, epiandrosterone sulfate (*P* = 0.0076) was most associated with 2-year incidence of clinician-diagnosed DED. In addition, we found decreased glycerophosphocholines to be associated with DED, although not at metabolome-wide significance.

**Conclusions:**

This hypothesis-free metabolomic approach found decreased serum androgens to be highly associated with DED and adds important evidence to the growing body of research that links androgens to ocular surface disease and DED.

Dry eye disease (DED) is a multifactorial disease and has a complex etiology and pathophysiology.^1^ Dry eye disease has been associated with a wide range of traits, including systemic and metabolic conditions and dysfunctions, such as vitamin A deficiency, lower intake of omega-3 and omega-6 fatty acids, ovarian dysfunction, menopause, acne, diabetes mellitus, sarcoidosis, and use of systemic medications such as antihistamines, β-blockers, antidiuretics, and antidepressants.^2^ Despite the abundance of associations, little is known about the molecular basis of DED.

Since the advent of the omics era (genomics, transcriptomics, proteomics), our understanding of complex diseases has dramatically increased. Metabolomics is a recent addition to these techniques and has emerged as a powerful tool in biological research. It allows the simultaneous analysis of hundreds of metabolites from a biological sample and thus provides a snapshot of the metabolomic state of a tissue.^3^, ^4^ Metabolomics has the potential to identify biomarkers and functional pathways of disease and therefore can lead to new treatment options. The power of metabolomics as an intermediate phenotype in the analysis of complex traits has been demonstrated in many studies, including in ophthalmology.^5^

Given the many associations of DED with systemic and metabolic traits, this study aimed to explore the relationship between DED and serum metabolites using a hypothesis-free or nontargeted metabolomics approach in a large population-representative sample.

## Methods

### Study Sample

A total of 2819 subjects from the TwinsUK cohort for whom both metabolomic and phenotypic information were available were included. This cohort has been ascertained from the general population of the United Kingdom through national media campaigns.^6^ The TwinsUK cohort is largely female because its initial focus was on osteoporosis and only women were recruited. However, it has recruited men and women for the past 15 years, but there is a strong female volunteer bias, common to all twin cohorts. Twins from this registry have been shown to be comparable to age-matched general population singletons for a broad variety of medical and behavioral traits.^7^ Local ethics committee approval was obtained for the study, and volunteers gave informed consent. The research followed the tenets of the Declaration of Helsinki.

### Dry Eye Disease Outcome Variables

Subjects from the TwinsUK cohort were invited to complete a postal questionnaire in 2013. This included, among many questions about systemic disease and psychologic and other traits, the Short Questionnaire for Dry Eye Syndrome (SQDES), as used and validated in the Women's Health Study and Physicians' Health Study.^8^ The SQDES includes 2 symptom questions: (1) “How often do your eyes feel dry (not wet enough)?” and (2) “How often do your eyes feel irritated?” (with the possible answers being 0 = never, 1 = sometimes, 2 = often, or 3 = constantly), and a third question about a previous diagnosis of DED: (3) “Have you ever been diagnosed (by a clinician) as having dry eye syndrome?” (with the possible answers being yes or no). A subject was considered as having DED if there was the presence of both dryness and irritation constantly or often, and/or a report of a previous clinical diagnosis of DED. This definition was used as the primary outcome variable of our metabolomics analysis. Second, we analyzed symptoms (using the sum score of the 2 symptom questions of SQDES) and the presence of a clinical diagnosis separately. Subsequently, we checked significant metabolites for an association with incident cases of DED as defined by a new clinical diagnosis between 2011 and 2013 compared with controls without a clinical diagnosis of DED in both 2011 and 2013. Data on the SQDES were not collected in 2011.

### Metabolomics

Nontargeted ultrahigh-performance liquid chromatography and mass spectrometry were performed on fasting plasma samples of TwinsUK participants using the Metabolon platform (Metabolon, Inc., Durham, NC), as described previously in more detail.^9^ The metabolomic data set contained acylcarnitines, amino acids, carbohydrates, glycerophospholipids, lipids, nucleotides, peptides, sphingomyelins, steroids, vitamins, and xenobiotics.

### Statistical Association Analyses

Raw metabolomic data were median normalized for daily fluctuations of the method. Subsequently, because the data were not normally distributed, they were inverse normalized. To avoid bias of the parameter estimates arising from small sample size, metabolites determined in fewer than 2000 subjects were excluded. This led to a total of 341 serum metabolites determined, of which 222 were fully characterized metabolites at the time of analysis, and these were examined for an association with DED.

We used a linear mixed effect analysis to perform analyses of the relationship between DED and single metabolites, adjusting for age at the time when the questionnaire was administered, as well as age at venipuncture, body mass index (BMI), and sex (LME4 package in R). Additional fixed effects terms included family twin relatedness, batch, and zygosity. Ordinal variables, such as the dryness and irritation symptoms sum score, were analyzed as quantitative; for binary outcomes, such as a clinical diagnosis of DED, the switch “binomial” in the R LME4 package was used. *P* values were calculated with the analysis of variance function by likelihood ratio test of the full model including the metabolite versus the null model excluding the metabolite.

Because of the correlations between metabolites, principal component analysis was used to determine the number of principal components that were linearly uncorrelated. A Bonferroni correction using the number of principal components with an eigenvalue higher than 1 was used to correct for multiple testing. This led us to consider a *P* value of 0.0012 (0.05 divided by 43 principal components) or below statistically significant in the main analyses.

## Results

The majority of 2819 subjects in this study were female (n = 2652, 94.1%). The subjects had a mean age of 57 years (standard deviation [SD], 12.0 years), ranging from 17 to 82 years. The BMI ranged from 16 to 53 kg/m^2^, with a mean BMI of 26.1 kg/m^2^ (SD, 4.7 kg/m^2^).

Prevalence of DED, as defined by the SQDES, was 15.5% (n = 436). A total of 373 subjects (13.2%) had a clinical diagnosis of dry eye, and 183 subjects (6.5%) had both dryness and irritation symptoms often or constantly. Mean dryness score was 0.51 (SD, 0.70), and mean irritation score was 0.78 (SD, 0.64). In the 2-year period between 2011 and 2013, there were 120 incident cases (5.1%) with a clinical diagnosis of DED among 2374 subjects who completed the questionnaires in both years.

Of the 222 metabolites, 2 androgens reached a metabolome-wide significant association with DED, as defined by the SQDES. These metabolites were androsterone sulfate (*P* = 0.00030) and epiandrosterone sulfate (*P* = 0.00036). The 15 most-associated metabolites are listed in Table 1. In total, 5 androgens were included in the metabolomics screen, and they were the most associated metabolites. The next 10 strongest associations included 4 glycerophosphocholines, although not at metabolome-wide statistical significance.

By subdividing our metabolomic analysis into symptoms and a clinical diagnosis separately, stronger associations were found with symptoms (Table 2), showing highly, metabolome-wide, significant associations for all the 5 androgen metabolites. Similar metabolome-wide significant associations as the primary analysis were found analyzing a clinical diagnosis of DED (Table 3), with epiandrosterone sulfate (*P* = 0.00029) and androsterone sulfate (*P* = 0.00110) most associated. A complete overview of all association results of the 222 metabolites with the 3 outcome measures can be found in Supplemental Tables S1 to S3 (available at www.aaojournal.org).

The androgen metabolites were subsequently tested for an association with incidence of DED, and epiandrosterone sulfate (*P* = 0.0076) appeared to be the most strongly associated, with the other 4 androgens showing nominal significance levels, before multiple testing correction: 4-androsten-3beta,17beta-diol disulfate 1, *P* = 0.046; androsterone sulfate, *P* = 0.055; dehydroepiandrosterone sulfate, *P* = 0.063; and 4-androsten-3beta,17beta-diol disulfate 2, *P* = 0.085. These results suggest that epiandrosterone sulfate might be the most potent biomarker of DED.

**Figure 1:**
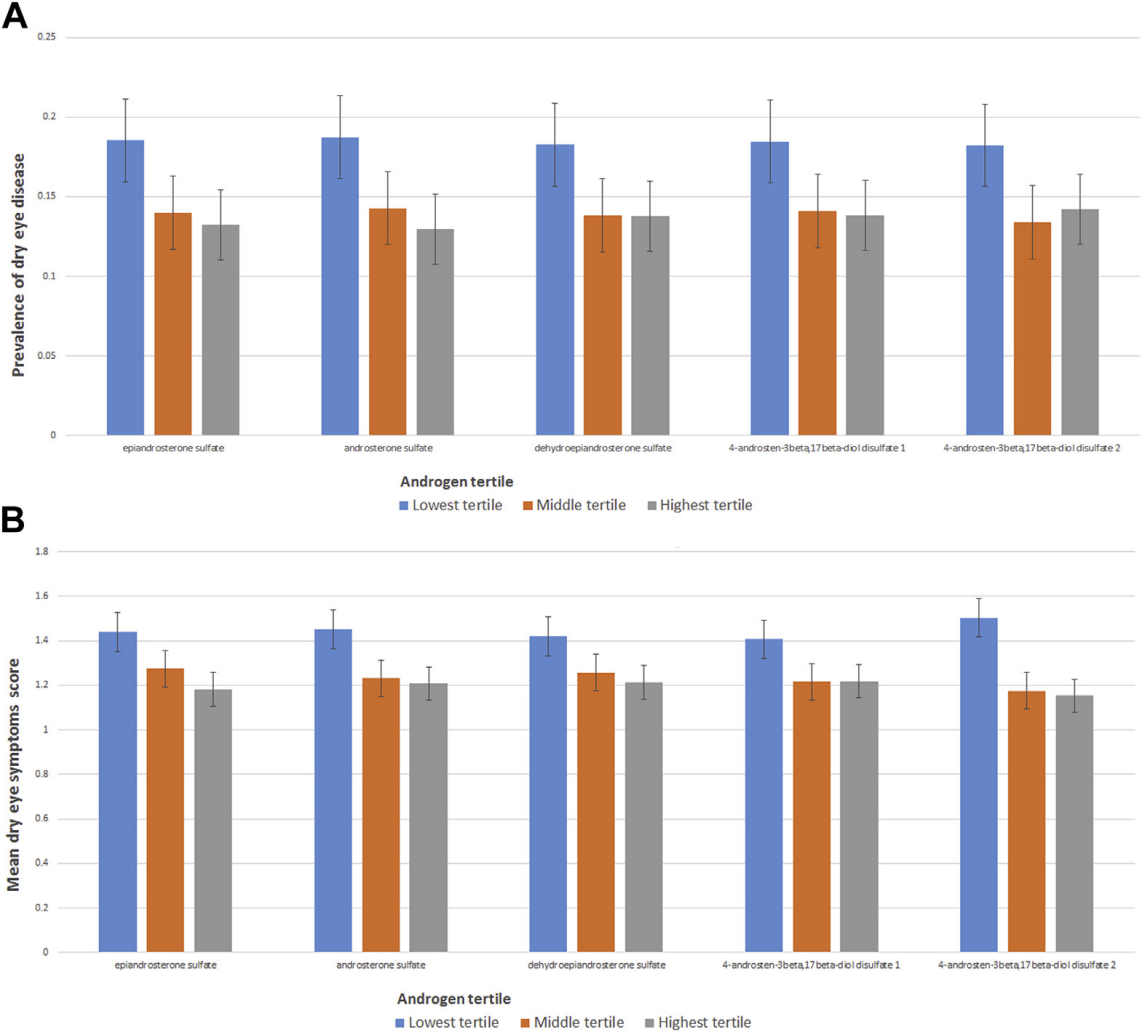
**A**, Prevalence of dry eye disease per serum androgen tertile. A subject was considered as having dry eye disease if there was presence of both dryness and irritation symptoms either constantly or often, and/or a report of a previous clinical diagnosis of dry eye disease. Serum androgen levels were corrected for age, body mass index (BMI) and gender. Error bars represent 95% confidence intervals. **B**, Dry eye symptoms score per serum androgen tertile. Dry eye symptoms score was the sum of 2 questions investigating the frequency of dryness symptoms and of irritation symptoms (both scored from 0 to 3). Serum androgen level were corrected for age, BMI and gender. Error bars represent 95% confidence intervals.

As expected,^10^ androgens declined with age in women (epiandrosterone sulfate β = −0.019, androsterone sulfate β = −0.020, DHEA-S β = −0.032, 4-androsten-3beta,17beta-diol disulfate 1 β = −0.021, 4-androsten-3beta,17beta-diol disulfate 2 β = −0.026, all *P* < 0.001). In men this decline with age was less strong for some of the androgens (epiandrosterone sulfate β = −0.008, *P* = 0.10; androsterone sulfate β = −0.018, *P* = 0.002; DHEA-S β = −0.033, *P* < 0.001, 4-androsten-3beta,17beta-diol disulfate 1 β = −0.014, *P* = 0.02, 4-androsten-3beta,17beta-diol disulfate 2 β = −0.012, *P* = 0.03). As the regression coefficients of the heavily transformed metabolites do not give easy interpretable information, Figure 1 gives an idea of the strength of the relationship between serum androgens (divided in 3 groups, based on their androgen levels, corrected for age, gender, and BMI) and prevalence of DED as defined by the SQDES (Fig 1A) and mean symptoms score (Fig 1B).

## Discussion

In this hypothesis-free study, we have shown strong associations of DED with all 5 androgens included in this screen of 222 known metabolites. The most significant associations with androgens were found with symptoms of DED, compared with a clinical diagnosis of DED.

Androgens, together with estrogens and progestogens, are the 3 sex steroids. They are released into the bloodstream after production in the testes and to a lesser degree in the ovaries and adrenal glands. In addition, sex steroids are produced by intracrine conversion of steroid precursors in other body tissues, such as body fat and skin. The biosynthetic pathway and interplay between androgens are complicated. Dehydroepiandrosterone (DHEA) is the precursor of androgens (and estrogens) and leads via enzymes such as 5α-reductase to epiandrosterone, androsterone, testosterone, and other steroids; 4-androstene-3beta,17beta-diol disulfate 1 and 2 also are known as androstenediols and are intermediates in the synthesis of testosterone from DHEA. Dehydroepiandrosterone sulfate (DHEA-S) is a metabolite of DHEA, but also can be back-converted to DHEA.^11^ Testosterone and di-hydro testosterone have the highest potency as androgens, and DHEA, epiandrosterone, and androsterone are relatively weak androgens. The androgens in our metabolomics screen are, not surprisingly, highly correlated, with correlations among the 5 androgens ranging from 0.55 to 0.94 (all *P* < 0.001).

This study is the first hypothesis-free study that links androgens to DED, but not the first overall study that links them. Androgen levels have been shown to influence the structure and function of the lacrimal and meibomian glands, with reduced androgens levels leading to reduced tear volume, reduced tear film stability by decreased quality and quantity of meibomian gland lipids, decreased tear turnover rate, and hyperosmolarity. In addition, androgens have been shown to have a direct effect on the tissues of the ocular surface, such as the conjunctiva, leading to altered mucin production.^12^, ^13^, ^14^, ^15^, ^16^ Also, women with Sjögren's disease have been shown to be androgen deficient.^17^ Although our metabolomic study is not testing causality, on the basis of all these studies, it is likely that reduced serum androgen levels are causative of DED. The exact relationship between serum androgens and tear film and ocular surface androgens remains to be elucidated. Glucuronide and sulfate androgen metabolites in the serum, as measured by our metabolomics screen, have been shown to be the most valid and possibly only reliable estimate of the total androgen pool including the intracrine production in peripheral tissues,^10^, ^18^ which is most likely the most important source in ocular surface tissues.

Given the results in this study, androgens might be a potential pathway for the treatment of DED. Bizzarro et al^19^ showed that 3 patients with DED and Sjögren syndrome had significantly increased Schirmer tests and decreased Bijsterveld scores after 60 days of oral testosterone undecanoate treatment, while no differences were found with respect to basal values after 60 days of placebo treatment. However, a recent, somewhat bigger, randomized, placebo-controlled pilot study on dry eye in 40 postmenopausal women did not show a beneficial effect of either transdermal testosterone, estrogen or both testosterone and estrogen on dry eye symptoms as compared to placebo.^20^ It did show a positive effect on tear secretion in the group with both testosterone cream and estrogen gel. In addition, in the testosterone group changes in serum 3-α-androstanediol-glucoronide, an androgen metabolite, were strongly positively associated with a change in tear breakup time (ρ = 0.83, *P* = 0.01), supporting our finding of an association between serum androgen metabolites and dry eye. In addition to these small randomized clinical trials there are also several case-series and case-reports reported that show a potential benefit of androgen treatment in dry eye disease. Among them, Nanavaty et al^21^ published a case series of 14 women with evaporative dry eye that significantly improved after use of transdermal androgen patches. Symptoms, measured by the Ocular Surface Disease Index, and signs, measured by tear breakup time and the Schirmer test, both improved after 3 weeks of androgen patching.^21^ Another case report reported improvement in DED signs and symptoms in a patient who received testosterone cream applied to the eyelids for 3 months.^22^ Other retrospective case studies reported improvement in DED after combined androgen and estrogen systemic therapy^23^ and worsening of DED parameters after antiandrogen therapy,^24^ but other studies failed to prove improvement of DED parameters after systemic DHEA supplementation in patients with Sjögren's syndrome.^25^, ^26^ Larger placebo-controlled studies are needed to further elucidate the possible effects of androgen treatment and to investigate which treatment modality is most useful and which patient groups are most likely to benefit.

A metabolomic approach to DED has been used before in a few settings, albeit always with a limited sample size and a small range of metabolites, but to our knowledge never using serum samples in a large population-based study. Galbis-Estrada et al^27^ performed a case-control study involving 90 participants with no, mild, or moderate DED and including 42 tear metabolites. The authors found a differential tear metabolomics profile among the 3 groups that decreased after supplementation with antioxidants and omega 3 fatty acids.^28^ The authors concluded that supplementation leads to restored normal tear profiles. No steroid metabolites were included in that study. A metabolomics analysis of human conjunctival epithelial cells in response to hyperosmotic stress found that levels of 21 metabolites significantly changed under hyperosmotic stress^29^; glycerophosphocholine increased most under hyperosmotic stress, and the authors concluded that this metabolite may act as an important osmoprotectant. Of note, in our primary analysis (Table 1), 4 of the 15 strongest metabolomic associations were glycerophosphocholines, with decreased levels associated with DED. The metabolite 1-palmitoylglycerophosphocholine also showed a significant association with incidence of a clinical diagnosis of DED (*P* = 0.020). So, this metabolite may act as a *serum* biomarker for dry eye. Our findings extend to these tear metabolomics studies showing that DED is associated not only with altered local metabolites but also with altered systemic metabolites. Our group previously showed that DED shares genetic factors with chronic widespread musculoskeletal pain.^30^ Of note, epiandrosterone sulfate levels were found to be inversely associated with chronic widespread musculoskeletal pain in a metabolomics study.^31^ Given these findings, one might speculate that the androgen pathway is a common mechanism to both these conditions. More studies are needed to investigate this in further detail.

### Study Limitations

First, despite the large sample size, this study was conducted on a single sample of volunteer twins and has not been independently replicated. Second, the majority of our subjects were female, potentially advantageous in a trait such as DED, which is more common in women, but the results may be less generalizable to men. Although our study included only 167 men, the direction of effect was the same for the androgen metabolites but seemed less strong. Defining exact menopausal status is difficult in population studies of this age range, but the effects were broadly similar in women below 55 years and above 55 years of age (data not shown). Third, the variable symptom sum score is semiquantitative, and analyzing it using regression-based techniques is imperfect given the assumptions they make. The advantages of our study include the large sample size compared with all previous studies in this field, the nontargeted nature of the metabolites measured, and the significant associations with clinical end points, corrected for multiple testing.

In summary, this hypothesis-free metabolomic study found highly significant associations between decreased androgen levels and DED, especially symptoms. Androgens, and possibly epiandrosterone the most, may act as a biomarker for dry eye, and we encourage more clinical trials to investigate whether topical or systemic androgen therapy is useful in DED. In addition, this study added evidence to the recent finding that glycerophosphocholines may be important as an osmoprotectant in DED.

## Figures and Tables

**Table 1:** Fifteen Strongest Associations of a Serum Metabolomics Study of Dry Eye Disease, as Defined by the Short Questionnaire of Dry Eye Disease†

Metabolite	Pathway	Super-pathway	*P* Value	β
Androsterone sulfate	Sterol/steroid	Lipid	0.00030∗	−0.20
Epiandrosterone sulfate	Sterol/steroid	Lipid	0.00036∗	−0.20
4-Androsten-3beta,17beta-diol disulfate 1	Sterol/steroid	Lipid	0.0023	−0.19
DHEA-S	Sterol/steroid	Lipid	0.0023	−0.18
4-Androsten-3beta,17beta-diol disulfate 2	Sterol/steroid	Lipid	0.0077	−0.17
Theophylline	Xanthine metabolism	Xenobiotics	0.016	0.14
N1-methyladenosine	Purine metabolism, adenine containing	Nucleotide	0.016	−0.13
1-Palmitoylglycerophosphocholine	Lysolipid	Lipid	0.022	−0.12
Caffeine	Xanthine metabolism	Xenobiotics	0.034	0.12
1,7-Dimethylurate	Xanthine metabolism	Xenobiotics	0.037	0.12
Serine	Glycine, serine, and threonine metabolism	Amino acid	0.046	0.11
2-Palmitoylglycerophosphocholine	Lysolipid	Lipid	0.050	−0.10
2-Linoleoylglycerophosphocholine	Lysolipid	Lipid	0.062	−0.11
1-Stearoylglycerophosphocholine	Lysolipid	Lipid	0.063	−0.10
Alanine	Alanine and aspartate metabolism	Amino acid	0.078	0.10

DHEA-S = dehydroepiandrosterone sulfate.

∗ Metabolome-wide significant association (*P* < 0.0012).

† A subject was considered as having dry eye disease (DED) if both dryness and irritation symptoms were present constantly or often, and/or a report of a previous clinical diagnosis of DED.

**Table 2:** Fifteen Strongest Associations of a Serum Metabolomics Study with Outcome Variable Dryness and Irritation Symptoms

Metabolite	Pathway	Super-pathway	*P* Value	β
4-Androsten-3beta,17beta-diol disulfate 2	Sterol/steroid	Lipid	2.9E-08∗	−0.16
Androsterone sulfate	Sterol/steroid	Lipid	0.0000040∗	−0.11
Epiandrosterone sulfate	Sterol/steroid	Lipid	0.000016∗	−0.11
4-Androsten-3beta,17beta-diol disulfate 1	Sterol/steroid	Lipid	0.000064∗	−0.11
DHEA-S	Sterol/steroid	Lipid	0.00011∗	−0.10
3-(4-Hydroxyphenyl)lactate	Phenylalanine and tyrosine metabolism	Amino acid	0.0021	−0.07
Glycerol	Glycerolipid metabolism	Lipid	0.00394	0.07
Erythronate	Amino sugars metabolism	Carbohydrate	0.025	0.05
Heptanoate (7:0)	Medium-chain fatty acid	Lipid	0.027	0.05
Allantoin	Purine metabolism, urate metabolism	Nucleotide	0.028	0.06
1,7-Dimethylurate	Xanthine metabolism	Xenobiotics	0.031	0.06
Linolenate (α or γ [18:3n3 or 6])	Essential fatty acid	Lipid	0.031	0.05
1-Oleoylglycerophosphoethanolamine	Lysolipid	Lipid	0.036	0.05
Caproate (6:0)	Medium-chain fatty acid	Lipid	0.042	0.05
Linoleate (18:2n6)	Essential fatty acid	Lipid	0.043	0.05

DHEA-S = dehydroepiandrosterone sulfate.

∗ Metabolome-wide significant association (*P* < 0.0012).

**Table 3:** Fifteen Strongest Associations of a Serum Metabolomics Study with Outcome Variable a Clinical Diagnosis of Dry Eye Disease

Metabolite	Pathway	Super-pathway	*P* Value	β
Epiandrosterone sulfate	Sterol/steroid	Lipid	0.00029∗	−0.22
Androsterone sulfate	Sterol/steroid	Lipid	0.0011∗	−0.20
DHEA-S	Sterol/steroid	Lipid	0.0041	−0.18
4-Androsten-3beta,17beta-diol disulfate 1	Sterol/steroid	Lipid	0.014	−0.17
N1-methyladenosine	Purine metabolism, adenine containing	Nucleotide	0.017	−0.14
Serine	Glycine, serine, and threonine metabolism	Amino acid	0.026	0.13
1-Palmitoylglycerophosphocholine	Lysolipid	Lipid	0.030	−0.12
4-Androsten-3beta,17beta-diol disulfate 2	Sterol/steroid	Lipid	0.034	−0.14
5-Dodecenoate (12:1n7)	Medium-chain fatty acid	Lipid	0.048	−0.12
Xanthine	Purine metabolism, (hypo)xanthine/inosine containing	Nucleotide	0.051	−0.13
Theophylline	Xanthine metabolism	Xenobiotics	0.051	0.12
Myristoleate (14:1n5)	Long-chain fatty acid	Lipid	0.054	−0.12
Indolelactate	Tryptophan metabolism	Amino acid	0.057	−0.11
Myristate (14:0)	Long-chain fatty acid	Lipid	0.059	−0.12
1-Stearoylglycerophosphocholine	Lysolipid	Lipid	0.063	−0.11

DHEA-S = dehydroepiandrosterone sulfate.

∗ Metabolome-wide significant association (*P* < 0.0012).
