# Supplementary material for: A Metabolome-Wide Study of Dry Eye Disease Reveals Serum Androgens as Biomarkers
Source: Ophthalmology. 2017 Apr;124(4):505–11. doi: 10.1016/j.ophtha.2016.12.011 (PMC5375174; doi:10.1016/j.ophtha.2016.12.011)
Supplement: Supplemental Table S1 [file mmc1.pdf]

Supplemental Table S1: Complete association results of a serum metabolomics of dry eye disease[I], ordered by *P*-value.

Metabolite	Pathway	Super-pathway	P-value	Beta
androsterone sulfate	Sterol/Steroid	Lipid	0.000297316	-0.2041044
epiandrosterone sulfate	Sterol/Steroid	Lipid	0.000361852	-0.200962718
4-androsten-3beta,17beta-diol disulfate 1*	Sterol/Steroid	Lipid	0.002269213	-0.194854716
dehydroisoandrosterone sulfate (DHEA-S)	Sterol/Steroid	Lipid	0.002292265	-0.183184893
4-androsten-3beta,17beta-diol disulfate 2*	Sterol/Steroid	Lipid	0.007706761	-0.170728093
theophylline	Xanthine metabolism	Xenobiotics	0.015834425	0.136858493
N1-methyladenosine	Purine metabolism, adenine containing	Nucleotide	0.015994007	-0.131957949
1-palmitoylglycerophosphocholine	Lysolipid	Lipid	0.022084951	-0.115056477
caffeine	Xanthine metabolism	Xenobiotics	0.03414392	0.119333957
1,7-dimethylurate	Xanthine metabolism	Xenobiotics	0.037368642	0.123718145
serine	Glycine, serine and threonine metabolism	Amino acid	0.045928814	0.107144048
2-palmitoylglycerophosphocholine*	Lysolipid	Lipid	0.04960331	-0.10247175
2-linoleoylglycerophosphocholine*	Lysolipid	Lipid	0.06188007	-0.111619635
1-stearoylglycerophosphocholine	Lysolipid	Lipid	0.062772945	-0.098793907
alanine	Alanine and aspartate metabolism	Amino acid	0.077530092	0.095822933
paraxanthine	Xanthine metabolism	Xenobiotics	0.082859665	0.096549293
dimethylarginine (SDMA + ADMA)	Urea cycle; arginine-, proline-, metabolism	Amino acid	0.091851929	-0.092115118
glutaroyl carnitine	Lysine metabolism	Amino acid	0.094314986	-0.090797295
1-oleoylglycerophosphocholine	Lysolipid	Lipid	0.095911529	-0.085506307
1-linoleoylglycerophosphocholine	Lysolipid	Lipid	0.096647366	-0.087239888
3-(4-hydroxyphenyl)lactate	Phenylalanine & tyrosine metabolism	Amino acid	0.103865832	-0.087975507
4-acetamidobutanoate	Guanidino and acetamido metabolism	Amino acid	0.105887511	-0.099800894
cortisone	Sterol/Steroid	Lipid	0.108947555	-0.090315331
margarate (17:0)	Long chain fatty acid	Lipid	0.130789043	-0.082233552
glycochenodeoxycholate	Bile acid metabolism	Lipid	0.131173423	0.085727513
2-hydroxypalmitate	Fatty acid, monohydroxy	Lipid	0.134686294	-0.079234034
1-myristoylglycerophosphocholine	Lysolipid	Lipid	0.14078931	-0.078505588
citrulline	Urea cycle; arginine-, proline-, metabolism	Amino acid	0.142493558	-0.083696976
alpha-ketoglutarate	Krebs cycle	Energy	0.14269881	0.090091213
undecanoate (11:0)	Medium chain fatty acid	Lipid	0.149265523	-0.07792308
myristate (14:0)	Long chain fatty acid	Lipid	0.152353854	-0.081971551
creatinine	Creatine metabolism	Amino acid	0.156549545	-0.076132487
10-nonadecenoate (19:1n9)	Long chain fatty acid	Lipid	0.159163315	-0.07954789
5-dodecenoate (12:1n7)	Medium chain fatty acid	Lipid	0.159739927	-0.07776334

myristoleate (14:1n5)	Long chain fatty acid	Lipid	0.161910634	-0.078871062
methionine	Cysteine, methionine, SAM, taurine metabolism	Amino acid	0.166727918	0.078479352
glycerophosphorylcholine (GPC)	Glycerolipid metabolism	Lipid	0.167555302	-0.078791482
glycine	Glycine, serine and threonine metabolism	Amino acid	0.1713396	0.073328547
choline	Glycerolipid metabolism	Lipid	0.178565355	0.072535276
palmitoyl sphingomyelin	Sphingolipid	Lipid	0.192440424	-0.080677902
gamma-glutamyltyrosine	gamma-glutamyl	Peptide	0.198335937	-0.077684322
1-palmitoleoylglycerophosphocholine*	Lysolipid	Lipid	0.205070661	-0.067646853
2-stearoylglycerophosphocholine*	Lysolipid	Lipid	0.219916464	-0.064436571
2-aminobutyrate	Butanoate metabolism	Amino acid	0.236077011	-0.064707593
1-palmitoylglycerophosphoethanolamine	Lysolipid	Lipid	0.237909906	0.062416077
palmitoleate (16:1n7)	Long chain fatty acid	Lipid	0.239635327	-0.066599471
indolelactate	Tryptophan metabolism	Amino acid	0.240045421	-0.064673206
isobutyrylcarnitine	Valine, leucine and isoleucine metabolism	Amino acid	0.24610178	0.063408215
dihomo-linoleate (20:2n6)	Long chain fatty acid	Lipid	0.251643326	-0.064412292
allantoin	Purine metabolism, urate metabolism	Nucleotide	0.253306214	0.068771481
2-hydroxyisobutyrate	Valine, leucine and isoleucine metabolism	Amino acid	0.254684558	0.071524921
alpha-hydroxyisovalerate	Valine, leucine and isoleucine metabolism	Amino acid	0.255386888	-0.062180012
pentadecanoate (15:0)	Long chain fatty acid	Lipid	0.257203924	-0.062595616
xanthine	Purine metabolism, (hypo)xanthine/inosine containing	Nucleotide	0.259870178	-0.068468108
ursodeoxycholate	Bile acid metabolism	Lipid	0.260964675	0.069924953
10-heptadecenoate (17:1n7)	Long chain fatty acid	Lipid	0.266768624	-0.063076119
p-cresol sulfate	Phenylalanine & tyrosine metabolism	Amino acid	0.267065905	0.062069858
cortisol	Sterol/Steroid	Lipid	0.267892563	-0.063593704
isovalerate	Fatty acid metabolism	Lipid	0.271591391	0.072640652
erythronate*	Aminosugars metabolism	Carbohydrate	0.277850279	0.060427458
1-stearoylglycerol (1-monostearin)	Monoacylglycerol	Lipid	0.279527018	0.060358523
2-methylbutyroylcarnitine	Valine, leucine and isoleucine metabolism	Amino acid	0.281734697	0.062257852
trans-4-hydroxyproline	Urea cycle; arginine-, proline-, metabolism	Amino acid	0.286471774	0.058291759
theobromine	Xanthine metabolism	Xenobiotics	0.302571175	0.055390214
arachidonate (20:4n6)	Long chain fatty acid	Lipid	0.305774884	-0.054748292
1-stearoylglycerophosphoethanolamine	Lysolipid	Lipid	0.309890726	0.054819378
stachydrine	Food component/Plant	Xenobiotics	0.331345838	0.052734035
pro-hydroxy-pro	Dipeptide	Peptide	0.332923	0.050567832
1-arachidonoylglycerophosphoethanolamine*	Lysolipid	Lipid	0.333289847	0.048895312
tyrosine	Phenylalanine & tyrosine metabolism	Amino acid	0.33993471	-0.05419382
threonine	Glycine, serine and threonine metabolism	Amino acid	0.342973328	0.058814501

butyrylcarnitine	Fatty acid metabolism (also BCAA metabolism)	Lipid	0.343055131	0.053620785
N-acetylthreonine	Glycine, serine and threonine metabolism	Amino acid	0.343902303	0.054688076
acetylcarnitine	Carnitine metabolism	Lipid	0.34413242	-0.052897454
pyruvate	Glycolysis, gluconeogenesis, pyruvate metabolism	Carbohydrate	0.350074598	0.053558132
1-eicosadienoylglycerophosphocholine*	Lysolipid	Lipid	0.364921181	-0.051001882
eicosenoate (20:1n9 or 11)	Long chain fatty acid	Lipid	0.368934368	-0.050331998
cysteine	Cysteine, methionine, SAM, taurine metabolism	Amino acid	0.371055967	-0.047683242
decanoylcarnitine	Carnitine metabolism	Lipid	0.374862479	-0.050209885
pseudouridine	Pyrimidine metabolism, uracil containing	Nucleotide	0.379164413	-0.048865692
catechol sulfate	Benzoate metabolism	Xenobiotics	0.381844788	0.046626193
pantothenate	Pantothenate and CoA metabolism	Cofactors and vitamins	0.382664011	0.046475293
succinylcarnitine	Krebs cycle	Energy	0.387388928	-0.054715961
1,5-anhydroglucitol (1,5-AG)	Glycolysis, gluconeogenesis, pyruvate metabolism	Carbohydrate	0.389408807	-0.045416679
biliverdin	Hemoglobin and porphyrin metabolism	Cofactors and vitamins	0.397173051	-0.051045255
HWESASXX*	Polypeptide	Peptide	0.402866695	0.048877855
palmitate (16:0)	Long chain fatty acid	Lipid	0.412146927	-0.04659182
hyodeoxycholate	Bile acid metabolism	Lipid	0.420840746	-0.047669305
2-tetradecenoyl carnitine	Carnitine metabolism	Lipid	0.422460725	-0.046856517
1-stearoylglycerophosphoinositol	Lysolipid	Lipid	0.425164246	-0.043088218
cis-4-decenoyl carnitine	Carnitine metabolism	Lipid	0.425891939	-0.050806963
2-oleoylglycerophosphocholine*	Lysolipid	Lipid	0.432156011	-0.041830755
oleate (18:1n9)	Long chain fatty acid	Lipid	0.435377667	-0.043483298
1-palmitoylglycerophosphoinositol*	Lysolipid	Lipid	0.440142924	-0.04891335
1-eicosatrienoylglycerophosphocholine*	Lysolipid	Lipid	0.441440602	-0.038931048
1-heptadecanoylglycerophosphocholine	Lysolipid	Lipid	0.442377528	-0.040872259
laurate (12:0)	Medium chain fatty acid	Lipid	0.442422218	-0.042857211
threonate	Ascorbate and aldarate metabolism	Cofactors and vitamins	0.443054123	0.041031389
indolepropionate	Tryptophan metabolism	Amino acid	0.444877164	-0.040885887
histidine	Histidine metabolism	Amino acid	0.451318977	-0.041114872
gamma-glutamylphenylalanine	gamma-glutamyl	Peptide	0.451338865	-0.041262317
5-oxoproline	Glutathione metabolism	Amino acid	0.458763756	-0.04121392
bilirubin (Z,Z)	Hemoglobin and porphyrin metabolism	Cofactors and vitamins	0.460493211	0.043064972
glutamate	Glutamate metabolism	Amino acid	0.468719067	0.042395326
serotonin (5HT)	Tryptophan metabolism	Amino acid	0.470292753	-0.047866301
heptanoate (7:0)	Medium chain fatty acid	Lipid	0.47462611	-0.038418548
levulinate (4-oxovalerate)	Valine, leucine and isoleucine metabolism	Amino acid	0.476803062	0.038921477
isovalerylcarnitine	Valine, leucine and isoleucine metabolism	Amino acid	0.481173913	0.039750686
stearate (18:0)	Long chain fatty acid	Lipid	0.481600581	-0.040171806
3-methylhistidine	Histidine metabolism	Amino acid	0.482037753	-0.041399667

lysine	Lysine metabolism	Amino acid	0.484444516	0.037245064
1-docosahexaenoylglycerophosphocholine*	Lysolipid	Lipid	0.485829777	0.038117809
docosahexaenoate (DHA; 22:6n3)	Essential fatty acid	Lipid	0.501899068	0.037628006
hypoxanthine	Purine metabolism, (hypo)xanthine/inosine containing	Nucleotide	0.510205691	-0.038224626
ornithine	Urea cycle; arginine-, proline-, metabolism	Amino acid	0.510427648	-0.034782474
threitol	Nucleotide sugars, pentose metabolism	Carbohydrate	0.511490122	0.038612881
4-ethylphenylsulfate	Benzoate metabolism	Xenobiotics	0.512680318	0.037499288
3-phenylpropionate (hydrocinnamate)	Phenylalanine & tyrosine metabolism	Amino acid	0.5148475	0.039910953
trimethyl-N-aminovalerate	Carnitine metabolism	Lipid	0.517073763	0.036310775
linoleate (18:2n6)	Essential fatty acid	Lipid	0.522164138	-0.034797567
arginine	Urea cycle; arginine-, proline-, metabolism	Amino acid	0.527272102	0.03466151
taurochenodeoxycholate	Bile acid metabolism	Lipid	0.535689152	-0.039460286
glycerate	Glycolysis, gluconeogenesis, pyruvate metabolism	Carbohydrate	0.538677279	0.033129508
phenyllactate (PLA)	Phenylalanine & tyrosine metabolism	Amino acid	0.540400968	0.037629961
dihomo-linolenate (20:3n3 or n6)	Essential fatty acid	Lipid	0.542381167	-0.03188376
1-palmitoylplasmenylethanolamine*	Lysolipid	Lipid	0.544187917	-0.037045081
phosphate	Oxidative phosphorylation	Energy	0.546858576	-0.031925403
cholesterol	Sterol/Steroid	Lipid	0.552821624	-0.033403311
gamma-glutamylleucine	gamma-glutamyl	Peptide	0.553398009	0.036119125
piperine	Food component/Plant	Xenobiotics	0.556855156	-0.033952432
uridine	Pyrimidine metabolism, uracil containing	Nucleotide	0.557271735	-0.030194849
aspartate	Alanine and aspartate metabolism	Amino acid	0.558207828	-0.030913471
lathosterol	Sterol/Steroid	Lipid	0.564815923	-0.036490057
4-methyl-2-oxopentanoate	Valine, leucine and isoleucine metabolism	Amino acid	0.571915286	-0.033430175
nonadecanoate (19:0)	Long chain fatty acid	Lipid	0.572447166	-0.031327349
isoleucine	Valine, leucine and isoleucine metabolism	Amino acid	0.591227499	0.029550091
asparagine	Alanine and aspartate metabolism	Amino acid	0.591329543	0.029478063
pyroglutamine*	Glutamate metabolism	Amino acid	0.594283823	-0.030646401
scyllo-inositol	Inositol metabolism	Lipid	0.595463246	0.031837045
glycerol	Glycerolipid metabolism	Lipid	0.596711799	0.029691386
proline	Urea cycle; arginine-, proline-, metabolism	Amino acid	0.603548346	0.028376391
arabinose	Nucleotide sugars, pentose metabolism	Carbohydrate	0.625793402	-0.03072093
stearidonate (18:4n3)	Long chain fatty acid	Lipid	0.628066231	-0.025792191
creatine	Creatine metabolism	Amino acid	0.63244339	-0.02688376
1-arachidonoylglycerophosphoinositol*	Lysolipid	Lipid	0.645524614	0.025770567
7-methylxanthine	Xanthine metabolism	Xenobiotics	0.658463514	-0.028156381
3-indoxyl sulfate	Tryptophan metabolism	Amino acid	0.667442924	0.022771236

bilirubin (E,E)*	Hemoglobin and porphyrin metabolism	Cofactors and vitamins	0.672040382	0.024658998
palmitoylcarnitine	Carnitine metabolism	Lipid	0.676688125	-0.023219917
4-vinylphenol sulfate	Benzoate metabolism	Xenobiotics	0.680986633	0.023366158
dodecanedioate	Fatty acid, dicarboxylate	Lipid	0.682616841	0.022731036
heme*	Hemoglobin and porphyrin	Cofactors and vitamins	0.68780922	0.023514134
erythrose	Fructose, mannose, galactose, starch, and sucrose metabolism	Carbohydrate	0.687943887	-0.022584289
C-glycosyltryptophan*	Tryptophan metabolism	Amino acid	0.695265726	-0.024005052
erythritol	Sugar, sugar substitute, starch	Xenobiotics	0.696231609	0.021828683
1-palmitoylglycerol (1-monopalmitin)	Monoacylglycerol	Lipid	0.701581816	-0.020542877
glycerol 2-phosphate	Chemical	Xenobiotics	0.704724451	0.023547074
linolenate [alpha or gamma; (18:3n3 or 6)]	Essential fatty acid	Lipid	0.712534296	0.021660618
N-acetylornithine	Urea cycle; arginine-, proline-, metabolism	Amino acid	0.714668109	-0.020059166
stearoylcarnitine	Carnitine metabolism	Lipid	0.720504178	-0.021398213
quinate	Food component/Plant	Xenobiotics	0.721750633	0.021173345
urate	Purine metabolism, urate metabolism	Nucleotide	0.724171041	0.021194773
10-undecenoate (11:1n1)	Medium chain fatty acid	Lipid	0.740205551	-0.018264575
N-acetylalanine	Alanine and aspartate metabolism	Amino acid	0.742052382	-0.017810098
3-hydroxybutyrate (BHBA)	Ketone bodies	Lipid	0.74893887	-0.017417759
betaine	Glycine, serine and threonine metabolism	Amino acid	0.752950889	0.01793887
glycocholate	Bile acid metabolism	Lipid	0.758473539	0.018140113
adrenate (22:4n6)	Long chain fatty acid	Lipid	0.762734295	0.016065189
tryptophan	Tryptophan metabolism	Amino acid	0.764912482	-0.016501241
tetradecanedioate	Fatty acid, dicarboxylate	Lipid	0.768371863	-0.017098768
glucose	Glycolysis, gluconeogenesis, pyruvate metabolism	Carbohydrate	0.771020673	0.015667547
gamma-glutamylvaline	gamma-glutamyl	Peptide	0.781749195	0.017419478
1-oleoylglycerophosphoethanolamine	Lysolipid	Lipid	0.79209251	0.014141893
pelargonate (9:0)	Medium chain fatty acid	Lipid	0.798105093	-0.014266462
alpha-tocopherol	Tocopherol metabolism	Cofactors and vitamins	0.801781147	-0.015678054
propionylcarnitine	Fatty acid metabolism (also BCAA metabolism)	Lipid	0.810326606	0.013997157
mannose	Fructose, mannose, galactose, starch, and sucrose metabolism	Carbohydrate	0.813625279	-0.013218581
malate	Krebs cycle	Energy	0.814050246	-0.013495514
octadecanedioate	Fatty acid, dicarboxylate	Lipid	0.817037034	-0.013361316
indoleacetate	Tryptophan metabolism	Amino acid	0.819213184	-0.011903944
octanoylcarnitine	Carnitine metabolism	Lipid	0.822925222	-0.012250609
3-methyl-2-oxobutyrate	Valine, leucine and isoleucine metabolism	Amino acid	0.823789309	-0.012951928
oleoylcarnitine	Carnitine metabolism	Lipid	0.823828058	-0.012095744
docosapentaenoate (n3 DPA; 22:5n3)	Essential fatty acid	Lipid	0.829106224	-0.011922278

2-hydroxystearate	Fatty acid, monohydroxy	Lipid	0.835045167	-0.010533662
leucine	Valine, leucine and isoleucine metabolism	Amino acid	0.835382886	0.011861082
phenol sulfate	Phenylalanine & tyrosine metabolism	Amino acid	0.846037703	0.009714127
pipecolate	Lysine metabolism	Amino acid	0.846887905	0.010504288
1-arachidonoylglycerophosphocholine*	Lysolipid	Lipid	0.84753598	-0.009723492
N-acetylglycine	Glycine, serine and threonine metabolism	Amino acid	0.847774167	-0.011619825
kynurenine	Tryptophan metabolism	Amino acid	0.854307341	0.009924531
3-carboxy-4-methyl-5-propyl-2-furanpropanoate (CMPF)	Fatty acid, dicarboxylate	Lipid	0.854388077	0.010305839
phenylalanine	Phenylalanine & tyrosine metabolism	Amino acid	0.856336154	-0.009501841
tryptophan betaine	Tryptophan metabolism	Amino acid	0.857933466	-0.011421185
phenylacetylglutamine	Phenylalanine & tyrosine metabolism	Amino acid	0.86083473	-0.009342407
gamma-tocopherol	Tocopherol metabolism	Cofactors and vitamins	0.862594691	0.010477243
pyridoxate	Vitamin B6 metabolism	Cofactors and vitamins	0.869814223	0.008249855
urea	Urea cycle; arginine-, proline-, metabolism	Amino acid	0.882521425	0.009222705
2-hydroxybutyrate (AHB)	Cysteine, methionine, SAM, taurine metabolism	Amino acid	0.883011116	0.008556371
beta-hydroxyisovalerate	Valine, leucine and isoleucine metabolism	Amino acid	0.887061962	-0.00747623
acetylphosphate	Oxidative phosphorylation	Energy	0.889691872	0.007592362
eicosapentaenoate (EPA; 20:5n3)	Essential fatty acid	Lipid	0.889978048	0.007883795
citrate	Krebs cycle	Energy	0.890338893	-0.008412569
hexadecanedioate	Fatty acid, dicarboxylate	Lipid	0.898356068	-0.007673929
carnitine	Carnitine metabolism	Lipid	0.901492565	0.006912125
glutamine	Glutamate metabolism	Amino acid	0.90243775	-0.00713302
hexanoylcarnitine	Carnitine metabolism	Lipid	0.909772933	-0.006272453
caproate (6:0)	Medium chain fatty acid	Lipid	0.911672791	0.005958997
hippurate	Benzoate metabolism	Xenobiotics	0.915795835	0.005938247
taurolithocholate 3-sulfate	Bile acid metabolism	Lipid	0.937177972	-0.00532227
1-linoleoylglycerophosphoethanolamine*	Lysolipid	Lipid	0.93745325	0.003959016
valine	Valine, leucine and isoleucine metabolism	Amino acid	0.941161477	-0.003527071
fructose	Fructose, mannose, galactose, starch, and sucrose metabolism	Carbohydrate	0.941174988	-0.004596127
glycerol 3-phosphate (G3P)	Glycerolipid metabolism	Lipid	0.94341736	-0.003083935
lactate	Glycolysis, gluconeogenesis, pyruvate metabolism	Carbohydrate	0.950458279	0.003481271
caprylate (8:0)	Medium chain fatty acid	Lipid	0.953243953	-0.003834028
cholate	Bile acid metabolism	Lipid	0.957583023	0.003431784
7-alpha-hydroxy-3-oxo-4-cholestenoate (7-Hoca)	Sterol/Steroid	Lipid	0.983701322	0.000653765
gamma-glutamylglutamine	gamma-glutamyl	Peptide	0.988870552	0.000843416
benzoate	Benzoate metabolism	Xenobiotics	0.989528191	-0.000725936
3-methyl-2-oxovalerate	Valine, leucine and isoleucine metabolism	Amino acid	0.991396295	-0.000545361

myo-inositol	Inositol metabolism	Lipid	1	0.000648873

ǂA subject was considered as having dry eye disease if there was presence of both dryness and irritation symptoms either constantly or often, and/or a report of a previous clinical diagnosis of dry eye disease.
