# Supplementary material for: A Metabolome-Wide Study of Dry Eye Disease Reveals Serum Androgens as Biomarkers
Source: Ophthalmology. 2017 Apr;124(4):505–11. doi: 10.1016/j.ophtha.2016.12.011 (PMC5375174; doi:10.1016/j.ophtha.2016.12.011)
Supplement: Supplemental Table S2 [file mmc2.pdf]

Supplemental Table S2: Complete association results of a serum metabolomics study with outcome variable dryness and irritation symptoms, ordered by *P*-value.

Metabolite	Pathway	Super-pathway	P-value	Beta
4-androsten-3beta,17beta-diol disulfate 2*	Sterol/Steroid	Lipid	2.89E-08	-0.156457853
androsterone sulfate	Sterol/Steroid	Lipid	0.00000401	-0.112958237
epiandrosterone sulfate	Sterol/Steroid	Lipid	0.0000157	-0.106112773
4-androsten-3beta,17beta-diol disulfate 1*	Sterol/Steroid	Lipid	0.0000641	-0.112597307
dehydroepiandrosterone sulfate (DHEA-S)	Sterol/Steroid	Lipid	0.000113533	-0.101490182
3-(4-hydroxyphenyl)lactate	Phenylalanine & tyrosine metabolism	Amino acid	0.002105103	-0.073368453
glycerol	Glycerolipid metabolism	Lipid	0.003937188	0.069676049
erythronate*	Aminosugars metabolism	Carbohydrate	0.02488977	0.054207175
heptanoate (7:0)	Medium chain fatty acid	Lipid	0.027106437	0.051533273
allantoin	Purine metabolism, urate metabolism	Nucleotide	0.02821878	0.058169696
1,7-dimethylurate	Xanthine metabolism	Xenobiotics	0.031144017	0.056524589
linolenate [alpha or gamma; (18:3n3 or 6)]	Essential fatty acid	Lipid	0.031441255	0.051806801
1-oleoylglycerophosphoethanolamine	Lysolipid	Lipid	0.036388271	0.047298226
caproate (6:0)	Medium chain fatty acid	Lipid	0.041681723	0.047520952
linoleate (18:2n6)	Essential fatty acid	Lipid	0.042565811	0.049001082
caprylate (8:0)	Medium chain fatty acid	Lipid	0.045301506	0.047254214
10-heptadecenoate (17:1n7)	Long chain fatty acid	Lipid	0.046530063	0.049918098
phenol sulfate	Phenylalanine & tyrosine metabolism	Amino acid	0.05030169	0.045728954
trans-4-hydroxyproline	Urea cycle; arginine-, proline-, metabolism	Amino acid	0.050850146	0.045272664
1-arachidonoylglycerophosphoinositol*	Lysolipid	Lipid	0.056733674	0.044672193
theophylline	Xanthine metabolism	Xenobiotics	0.056978801	0.046921003
alpha-ketoglutarate	Krebs cycle	Energy	0.057445637	0.050316778
palmitoleate (16:1n7)	Long chain fatty acid	Lipid	0.06035818	0.047077701
pantothenate	Pantothenate and CoA metabolism	Cofactors and vitamins	0.061799215	0.04391447

ornithine	Urea cycle; arginine-, proline-, metabolism	Amino acid	0.066088399	-0.041609034
phosphate	Oxidative phosphorylation	Energy	0.073505005	0.041847262
cortisol	Sterol/Steroid	Lipid	0.075569705	-0.04292455
choline	Glycerolipid metabolism	Lipid	0.08268641	0.040756017
myristoleate (14:1n5)	Long chain fatty acid	Lipid	0.093724608	0.041643592
butyrylcarnitine	Fatty acid metabolism (also BCAA metabolism)	Lipid	0.093826337	0.041780486
urate	Purine metabolism, urate metabolism	Nucleotide	0.095898492	0.04253841
glycine	Glycine, serine and threonine metabolism	Amino acid	0.096300292	0.039335634
methionine	Cysteine, methionine, SAM, taurine metabolism	Amino acid	0.105132091	0.039366133
pelargonate (9:0)	Medium chain fatty acid	Lipid	0.10578073	0.038305285
myristate (14:0)	Long chain fatty acid	Lipid	0.108092465	0.040013303
caffeine	Xanthine metabolism	Xenobiotics	0.112517576	0.038886321
biliverdin	Hemoglobin and porphyrin metabolism	Cofactors and vitamins	0.112613458	-0.041466041
2-hydroxyisobutyrate	Valine, leucine and isoleucine metabolism	Amino acid	0.113047531	0.043567455
dimethylarginine (SDMA + ADMA)	Urea cycle; arginine-, proline-, metabolism	Amino acid	0.115004345	-0.037225927
2-aminobutyrate	Butanoate metabolism	Amino acid	0.120981871	-0.036670649
cholesterol	Sterol/Steroid	Lipid	0.124770269	0.03770056
2-linoleoylglycerophosphocholine*	Lysolipid	Lipid	0.125648408	-0.03882312
1-arachidonoylglycerophosphoethanolamine*	Lysolipid	Lipid	0.130816826	0.033534003
alanine	Alanine and aspartate metabolism	Amino acid	0.132939619	0.035243967
catechol sulfate	Benzoate metabolism	Xenobiotics	0.138003958	0.03506565
arginine	Urea cycle; arginine-, proline-, metabolism	Amino acid	0.140604654	0.035217626
piperine	Food component/Plant	Xenobiotics	0.142118671	-0.036083878

threitol	Nucleotide sugars, pentose metabolism	Carbohydrate	0.148347924	0.036808225
5-dodecenoate (12:1n7)	Medium chain fatty acid	Lipid	0.149920541	0.034813767
arabinose	Nucleotide sugars, pentose metabolism	Carbohydrate	0.150910999	-0.038882093
alpha-hydroxyisovalerate	Valine, leucine and isoleucine metabolism	Amino acid	0.153761354	-0.034273899
heme*	Hemoglobin and porphyrin	Cofactors and vitamins	0.159887691	0.035879571
oleate (18:1n9)	Long chain fatty acid	Lipid	0.163725552	0.034113705
palmitate (16:0)	Long chain fatty acid	Lipid	0.170999135	0.033644319
glutamate	Glutamate metabolism	Amino acid	0.173785343	0.034167627
pipecolate	Lysine metabolism	Amino acid	0.182621926	0.032023776
4-vinylphenol sulfate	Benzoate metabolism	Xenobiotics	0.184230196	0.032909947
levulinate (4-oxovalerate)	Valine, leucine and isoleucine metabolism	Amino acid	0.195313113	0.030118917
paraxanthine	Xanthine metabolism	Xenobiotics	0.205110569	0.030758603
cortisone	Sterol/Steroid	Lipid	0.21142851	-0.030368514
alpha-tocopherol	Tocopherol metabolism	Cofactors and vitamins	0.215695161	0.031907754
pro-hydroxy-pro	Dipeptide	Peptide	0.22101517	0.028387623
citrate	Krebs cycle	Energy	0.227954443	-0.029386023
1-palmitoylglycerophosphoethanolamine	Lysolipid	Lipid	0.248840203	0.02643905
1-linoleoylglycerophosphoethanolamine*	Lysolipid	Lipid	0.2512417	0.025792389
serine	Glycine, serine and threonine metabolism	Amino acid	0.252526872	0.026619431
N1-methyladenosine	Purine metabolism, adenine containing	Nucleotide	0.26073776	-0.026570542
undecanoate (11:0)	Medium chain fatty acid	Lipid	0.263897309	0.02597011
1-stearoylglycerol (1-monostearin)	Monoacylglycerol	Lipid	0.278251822	0.0263217
serotonin (5HT)	Tryptophan metabolism	Amino acid	0.282730205	0.029137924
quinate	Food component/Plant	Xenobiotics	0.290529098	0.028498437
glycerol 2-phosphate	Chemical	Xenobiotics	0.296833851	0.027336186
adrenate (22:4n6)	Long chain fatty acid	Lipid	0.299467085	0.023806739

threonine	Glycine, serine and threonine metabolism	Amino acid	0.301018632	0.028247454
indolepropionate	Tryptophan metabolism	Amino acid	0.31408542	-0.023584879
glycochenodeoxycholate	Bile acid metabolism	Lipid	0.316091018	0.024159502
mannose	Fructose, mannose, galactose, starch, and sucrose metabolism	Carbohydrate	0.323102058	-0.02389909
gamma-glutamyltyrosine	gamma-glutamyl	Peptide	0.331288456	-0.025779559
isovalerate	Fatty acid metabolism	Lipid	0.333186439	0.026411631
glutaroyl carnitine	Lysine metabolism	Amino acid	0.333885813	-0.022797585
4-methyl-2-oxopentanoate	Valine, leucine and isoleucine metabolism	Amino acid	0.334313636	-0.024367802
glycerol 3-phosphate (G3P)	Glycerolipid metabolism	Lipid	0.338438552	0.021691568
gamma-glutamylglutamine	gamma-glutamyl	Peptide	0.341204462	-0.02339507
tryptophan	Tryptophan metabolism	Amino acid	0.345465415	-0.021679206
1-palmitoylglycerophosphocholine	Lysolipid	Lipid	0.349333249	-0.020451864
3-phenylpropionate (hydrocinnamate)	Phenylalanine & tyrosine metabolism	Amino acid	0.351513274	0.024120981
benzoate	Benzoate metabolism	Xenobiotics	0.353464348	0.021439854
hexanoylcarnitine	Carnitine metabolism	Lipid	0.3544301	0.022780004
histidine	Histidine metabolism	Amino acid	0.357838613	0.022131095
laurate (12:0)	Medium chain fatty acid	Lipid	0.361056796	0.021970257
10-nonadecenoate (19:1n9)	Long chain fatty acid	Lipid	0.363352041	0.022342239
glycerate	Glycolysis, gluconeogenesis, pyruvate metabolism	Carbohydrate	0.371799218	0.021067406
stearoylcarnitine	Carnitine metabolism	Lipid	0.389173495	-0.021798344
1-linoleoylglycerophosphocholine	Lysolipid	Lipid	0.39207502	-0.019378179
N-acetylornithine	Urea cycle; arginine-, proline-, metabolism	Amino acid	0.394541864	-0.02025398
glutamine	Glutamate metabolism	Amino acid	0.396615795	0.020334493
phenyllactate (PLA)	Phenylalanine & tyrosine metabolism	Amino acid	0.402532654	-0.022670416
theobromine	Xanthine metabolism	Xenobiotics	0.409135803	0.019162045

malate	Krebs cycle	Energy	0.415719825	-0.019568889
7-methylxanthine	Xanthine metabolism	Xenobiotics	0.416707285	-0.021925986
pyridoxate	Vitamin B6 metabolism	Cofactors and vitamins	0.428181502	0.018614354
acetylphosphate	Oxidative phosphorylation	Energy	0.431972889	0.018200945
lathosterol	Sterol/Steroid	Lipid	0.433751589	0.02190005
citrulline	Urea cycle; arginine-, proline-, metabolism	Amino acid	0.437086734	-0.019033929
4-ethylphenylsulfate	Benzoate metabolism	Xenobiotics	0.439195468	0.019730093
1-stearoylglycerophosphoinositol	Lysolipid	Lipid	0.44763556	0.017659581
1-oleoylglycerophosphocholine	Lysolipid	Lipid	0.46005958	-0.016611961
isovalerylcarnitine	Valine, leucine and isoleucine metabolism	Amino acid	0.469018649	-0.017381441
hyodeoxycholate	Bile acid metabolism	Lipid	0.471314831	-0.01891697
beta-hydroxyisovalerate	Valine, leucine and isoleucine metabolism	Amino acid	0.474635789	0.016882565
docosapentaenoate (n3 DPA; 22:5n3)	Essential fatty acid	Lipid	0.47764182	0.017649077
tyrosine	Phenylalanine & tyrosine metabolism	Amino acid	0.503207556	-0.016495103
creatinine	Creatine metabolism	Amino acid	0.504686468	-0.015536941
cysteine	Cysteine, methionine, SAM, taurine metabolism	Amino acid	0.517722539	0.015057553
2-tetradecenoyl carnitine	Carnitine metabolism	Lipid	0.528188553	-0.015976425
2-hydroxypalmitate	Fatty acid, monohydroxy	Lipid	0.538472853	0.013935477
threonate	Ascorbate and aldarate metabolism	Cofactors and vitamins	0.538607484	0.01440131
xanthine	Purine metabolism, (hypo)xanthine/inosine containing	Nucleotide	0.543080738	-0.015665194
2-hydroxystearate	Fatty acid, monohydroxy	Lipid	0.548724013	0.013599068
dodecanedioate	Fatty acid, dicarboxylate	Lipid	0.555206643	0.014468271
cholate	Bile acid metabolism	Lipid	0.563338788	0.015042533
3-hydroxybutyrate (BHBA)	Ketone bodies	Lipid	0.572920208	0.013340236

p-cresol sulfate	Phenylalanine & tyrosine metabolism	Amino acid	0.577421029	0.013418433
stearate (18:0)	Long chain fatty acid	Lipid	0.580206489	0.013471354
tryptophan betaine	Tryptophan metabolism	Amino acid	0.581737686	-0.015213343
bilirubin (Z,Z)	Hemoglobin and porphyrin metabolism	Cofactors and vitamins	0.582914342	0.014246602
palmitoyl sphingomyelin	Sphingolipid	Lipid	0.589861218	0.014862477
taurolithocholate 3-sulfate	Bile acid metabolism	Lipid	0.592767806	0.013317187
valine	Valine, leucine and isoleucine metabolism	Amino acid	0.593283698	0.012568538
asparagine	Alanine and aspartate metabolism	Amino acid	0.603889922	0.012132429
trimethyl-N-aminovalerate	Carnitine metabolism	Lipid	0.608242798	0.012293212
indolelactate	Tryptophan metabolism	Amino acid	0.609027345	-0.012316775
proline	Urea cycle; arginine-, proline-, metabolism	Amino acid	0.617646228	0.011986937
C-glycosyltryptophan*	Tryptophan metabolism	Amino acid	0.619502555	0.013029919
pyruvate	Glycolysis, gluconeogenesis, pyruvate metabolism	Carbohydrate	0.624151144	0.011988989
1-palmitoleoylglycerophosphocholine*	Lysolipid	Lipid	0.626239728	0.011393959
7-alpha-hydroxy-3-oxo-4-cholestenoate (7-Hoca)	Sterol/Steroid	Lipid	0.626428693	0.011363333
pyroglutamine*	Glutamate metabolism	Amino acid	0.630720062	0.012130895
dihomo-linoleate (20:2n6)	Long chain fatty acid	Lipid	0.632871131	0.011690721
3-indoxyl sulfate	Tryptophan metabolism	Amino acid	0.633624805	0.011121922
propionylcarnitine	Fatty acid metabolism (also BCAA metabolism)	Lipid	0.643399103	0.011380731
2-palmitoylglycerophosphocholine*	Lysolipid	Lipid	0.649990767	-0.010286613
creatine	Creatine metabolism	Amino acid	0.650458644	0.011109857
gamma-glutamylphenylalanine	gamma-glutamyl	Peptide	0.653797019	-0.010789695
3-methyl-2-oxovalerate	Valine, leucine and isoleucine metabolism	Amino acid	0.668749421	-0.010613981
HWESASXX*	Polypeptide	Peptide	0.66885391	0.010886673

erythrose	Fructose, mannose, galactose, starch, and sucrose metabolism	Carbohydrate	0.676989345	0.009558762
1-myristoylglycerophosphocholine	Lysolipid	Lipid	0.686004412	-0.009353266
lysine	Lysine metabolism	Amino acid	0.688467689	0.009294329
bilirubin (E,E)*	Hemoglobin and porphyrin metabolism	Cofactors and vitamins	0.693287558	-0.009821971
dihomo-linolenate (20:3n3 or n6)	Essential fatty acid	Lipid	0.702709098	0.008875571
margarate (17:0)	Long chain fatty acid	Lipid	0.704596201	0.009025515
1-heptadecanoylglycerophosphocholine	Lysolipid	Lipid	0.717536388	0.008546542
acetylcarnitine	Carnitine metabolism	Lipid	0.722426521	-0.008591249
palmitoylcarnitine	Carnitine metabolism	Lipid	0.726698525	-0.008433654
carnitine	Carnitine metabolism	Lipid	0.729682635	0.008238981
1-palmitoylplasmenylethanolamine*	Lysolipid	Lipid	0.736177055	-0.008895685
isoleucine	Valine, leucine and isoleucine metabolism	Amino acid	0.739583933	0.007960969
glycerophosphorylcholine (GPC)	Glycerolipid metabolism	Lipid	0.740278297	-0.008223929
1-stearoylglycerophosphoethanolamine	Lysolipid	Lipid	0.741913814	0.007660921
scyllo-inositol	Inositol metabolism	Lipid	0.759537556	0.007776367
5-oxoproline	Glutathione metabolism	Amino acid	0.763643694	-0.007270488
taurochenodeoxycholate	Bile acid metabolism	Lipid	0.770039452	-0.007889676
1-arachidonoylglycerophosphocholine*	Lysolipid	Lipid	0.773710063	-0.006532771
eicosapentaenoate (EPA; 20:5n3)	Essential fatty acid	Lipid	0.779185388	0.006706841
1-palmitoylglycerophosphoinositol*	Lysolipid	Lipid	0.782122186	0.00738449
1-palmitoylglycerol (1-monopalmitin)	Monoacylglycerol	Lipid	0.788177333	-0.006203129
kynurenine	Tryptophan metabolism	Amino acid	0.788628549	0.006526953
succinylcarnitine	Krebs cycle	Energy	0.791300732	-0.007270961
ursodeoxycholate	Bile acid metabolism	Lipid	0.795353541	0.007169043
octadecanedioate	Fatty acid, dicarboxylate	Lipid	0.797465402	0.006311575
1-eicosadienoylglycerophosphocholine*	Lysolipid	Lipid	0.801808466	0.006168369
eicosenoate (20:1n9 or 11)	Long chain fatty acid	Lipid	0.802126557	-0.006143784
phenylacetylglutamine	Phenylalanine & tyrosine metabolism	Amino acid	0.80813789	0.005733632

erythritol	Sugar, sugar substitute, starch	Xenobiotics	0.81875158	0.005652444
2-oleoylglycerophosphocholine*	Lysolipid	Lipid	0.818826456	0.005371761
hexadecanedioate	Fatty acid, dicarboxylate	Lipid	0.830517131	0.005189027
gamma-glutamylvaline	gamma-glutamyl	Peptide	0.833371647	0.005500857
nonadecanoate (19:0)	Long chain fatty acid	Lipid	0.848401933	0.004643742
glycocholate	Bile acid metabolism	Lipid	0.849062751	0.004917798
N-acetylalanine	Alanine and aspartate metabolism	Amino acid	0.853153329	0.004442957
urea	Urea cycle; arginine-, proline-, metabolism	Amino acid	0.855033819	0.004611736
gamma-tocopherol	Tocopherol metabolism	Cofactors and vitamins	0.860358966	0.004520972
tetradecanedioate	Fatty acid, dicarboxylate	Lipid	0.867071059	0.004212792
uridine	Pyrimidine metabolism, uracil containing	Nucleotide	0.87047397	0.003808338
N-acetylthreonine	Glycine, serine and threonine metabolism	Amino acid	0.875546022	-0.003951552
stearidonate (18:4n3)	Long chain fatty acid	Lipid	0.877372777	0.003698204
leucine	Valine, leucine and isoleucine metabolism	Amino acid	0.88553179	0.00347746
1,5-anhydroglucitol (1,5-AG)	Glycolysis, gluconeogenesis, pyruvate metabolism	Carbohydrate	0.88883396	0.003275727
2-methylbutyroylcarnitine	Valine, leucine and isoleucine metabolism	Amino acid	0.89065836	0.003504567
docosahexaenoate (DHA; 22:6n3)	Essential fatty acid	Lipid	0.891446175	0.003314176
fructose	Fructose, mannose, galactose, starch, and sucrose metabolism	Carbohydrate	0.891657073	0.003101015
gamma-glutamylleucine	gamma-glutamyl	Peptide	0.8964108	0.003365708
hippurate	Benzoate metabolism	Xenobiotics	0.897561353	-0.003054962
1-eicosatrienoylglycerophosphocholine*	Lysolipid	Lipid	0.910003784	-0.002524681
1-stearoylglycerophosphocholine	Lysolipid	Lipid	0.911990684	-0.002547298

3-methyl-2-oxobutyrate	Valine, leucine and isoleucine metabolism	Amino acid	0.914701925	0.002671074
stachydrine	Food component/Plant	Xenobiotics	0.915619571	-0.002488631
2-stearoylglycerophosphocholine*	Lysolipid	Lipid	0.920504326	-0.002277895
2-hydroxybutyrate (AHB)	Cysteine, methionine, SAM, taurine metabolism	Amino acid	0.922278269	-0.002407243
pentadecanoate (15:0)	Long chain fatty acid	Lipid	0.926496605	0.002230508
hypoxanthine	Purine metabolism, (hypo)xanthine/inosine containing	Nucleotide	0.93076749	-0.00215609
lactate	Glycolysis, gluconeogenesis, pyruvate metabolism	Carbohydrate	0.931862287	0.002090325
oleoylcarnitine	Carnitine metabolism	Lipid	0.933074768	-0.00203911
decanoylcarnitine	Carnitine metabolism	Lipid	0.936282985	-0.001979502
phenylalanine	Phenylalanine & tyrosine metabolism	Amino acid	0.944070016	0.001656388
cis-4-decenoyl carnitine	Carnitine metabolism	Lipid	0.946433831	-0.001850358
4-acetamidobutanoate	Guanidino and acetamido metabolism	Amino acid	0.946599851	0.001754025
betaine	Glycine, serine and threonine metabolism	Amino acid	0.948607463	-0.001567674
glucose	Glycolysis, gluconeogenesis, pyruvate metabolism	Carbohydrate	0.952786466	0.001394911
aspartate	Alanine and aspartate metabolism	Amino acid	0.956143409	-0.001297755
3-methylhistidine	Histidine metabolism	Amino acid	0.956640194	0.001371583
arachidonate (20:4n6)	Long chain fatty acid	Lipid	0.961374702	0.001134838
indoleacetate	Tryptophan metabolism	Amino acid	0.973998267	-0.000756547
octanoylcarnitine	Carnitine metabolism	Lipid	0.975292684	0.000760718
pseudouridine	Pyrimidine metabolism, uracil containing	Nucleotide	0.982131887	0.000539096

isobutyrylcarnitine	Valine, leucine and isoleucine metabolism	Amino acid	0.98348493	0.00049283
10-undecenoate (11:1n1)	Medium chain fatty acid	Lipid	0.983768659	-0.000485292
3-carboxy-4-methyl-5-propyl-2-furanpropanoate (CMPF)	Fatty acid, dicarboxylate	Lipid	0.984451046	0.000472605
1-docosahexaenoylglycerophosphocholine*	Lysolipid	Lipid	0.985815093	0.000420415
myo-inositol	Inositol metabolism	Lipid	0.993135601	0.00020664
N-acetylglycine	Glycine, serine and threonine metabolism	Amino acid	0.99954432	-0.0000137
