# Supplementary material for: A Metabolome-Wide Study of Dry Eye Disease Reveals Serum Androgens as Biomarkers
Source: Ophthalmology. 2017 Apr;124(4):505–11. doi: 10.1016/j.ophtha.2016.12.011 (PMC5375174; doi:10.1016/j.ophtha.2016.12.011)
Supplement: Supplemental Table S3 [file mmc3.pdf]

Supplemental Table S3: Complete association results of a serum metabolomics study with outcome variable a clinical diagnosis of dry eye disease, ordered by *P*-value.

Metabolite	Pathway	Super-pathway	P-value	Beta
epiandrosterone sulfate	Sterol/Steroid	Lipid	0.000290781	-0.218742145
androsterone sulfate	Sterol/Steroid	Lipid	0.001104212	-0.197012358
dehydroepiandrosterone sulfate (DHEA-S)	Sterol/Steroid	Lipid	0.004142316	-0.184956199
4-androsten-3beta,17beta-diol disulfate 1*	Sterol/Steroid	Lipid	0.014212261	-0.167036805
N1-methyladenosine	Purine metabolism, adenine containing	Nucleotide	0.017275936	-0.13824799
serine	Glycine, serine and threonine metabolism	Amino acid	0.026238815	0.127350162
1-palmitoylglycerophosphocholine	Lysolipid	Lipid	0.03016996	-0.115627256
4-androsten-3beta,17beta-diol disulfate 2*	Sterol/Steroid	Lipid	0.034445636	-0.144149327
5-dodecenoate (12:1n7)	Medium chain fatty acid	Lipid	0.047602388	-0.117446655
xanthine	Purine metabolism, (hypo)xanthine/inosine containing	Nucleotide	0.050596437	-0.127492496
theophylline	Xanthine metabolism	Xenobiotics	0.051194421	0.117954641
myristoleate (14:1n5)	Long chain fatty acid	Lipid	0.053999497	-0.116881498
indolelactate	Tryptophan metabolism	Amino acid	0.056961617	-0.112295144
myristate (14:0)	Long chain fatty acid	Lipid	0.059435335	-0.115677335
1-stearoylglycerophosphocholine	Lysolipid	Lipid	0.062941142	-0.105528287
cortisone	Sterol/Steroid	Lipid	0.064647435	-0.11005104
4-acetamidobutanoate	Guanidino and acetamido metabolism	Amino acid	0.06558337	-0.120745941
3-(4-hydroxyphenyl)lactate	Phenylalanine & tyrosine metabolism	Amino acid	0.065787535	-0.105814394
10-nonadecenoate (19:1n9)	Long chain fatty acid	Lipid	0.067760925	-0.110282031
margarate (17:0)	Long chain fatty acid	Lipid	0.068897575	-0.10503999
glutaroyl carnitine	Lysine metabolism	Amino acid	0.078007201	-0.102140674
pseudouridine	Pyrimidine metabolism, uracil containing	Nucleotide	0.078431152	-0.103804979
caffeine	Xanthine metabolism	Xenobiotics	0.079084632	0.105777112

alanine	Alanine and aspartate metabolism	Amino acid	0.089776142	0.098233781
dimethylarginine (SDMA + ADMA)	Urea cycle; arginine-, proline-, metabolism	Amino acid	0.093910649	-0.09787477
1-oleoylglycerophosphocholine	Lysolipid	Lipid	0.094931291	-0.091224798
hyodeoxycholate	Bile acid metabolism	Lipid	0.096235159	-0.104247446
10-heptadecenoate (17:1n7)	Long chain fatty acid	Lipid	0.09713237	-0.101266698
creatinine	Creatine metabolism	Amino acid	0.102915811	-0.093017703
1,7-dimethylurate	Xanthine metabolism	Xenobiotics	0.104831172	0.103063908
palmitoleate (16:1n7)	Long chain fatty acid	Lipid	0.117018708	-0.095321855
pyruvate	Glycolysis, gluconeogenesis, pyruvate metabolism	Carbohydrate	0.118273434	0.096809683
gamma-glutamyltyrosine	gamma-glutamyl	Peptide	0.12114197	-0.100090497
undecanoate (11:0)	Medium chain fatty acid	Lipid	0.126904758	-0.087855618
methionine	Cysteine, methionine, SAM, taurine metabolism	Amino acid	0.134751188	0.090577756
alpha-ketoglutarate	Krebs cycle	Energy	0.135654691	0.098108444
arachidonate (20:4n6)	Long chain fatty acid	Lipid	0.144806365	-0.08333226
dihomo-linoleate (20:2n6)	Long chain fatty acid	Lipid	0.154770956	-0.085493566
paraxanthine	Xanthine metabolism	Xenobiotics	0.157509864	0.084085086
2-palmitoylglycerophosphocholine*	Lysolipid	Lipid	0.158996244	-0.077985304
2-hydroxypalmitate	Fatty acid, monohydroxy	Lipid	0.170787969	-0.077388357
1-linoleoylglycerophosphocholine	Lysolipid	Lipid	0.175627455	-0.075662268
laurate (12:0)	Medium chain fatty acid	Lipid	0.176852667	-0.080301739
choline	Glycerolipid metabolism	Lipid	0.186020283	0.075575013
pentadecanoate (15:0)	Long chain fatty acid	Lipid	0.200553692	-0.075716223
threonine	Glycine, serine and threonine metabolism	Amino acid	0.201824402	0.084285645
palmitoyl sphingomyelin	Sphingolipid	Lipid	0.202348651	-0.084106699
1-eicosadienoylglycerophosphocholine*	Lysolipid	Lipid	0.219428522	-0.07411916
oleate (18:1n9)	Long chain fatty acid	Lipid	0.22484671	-0.072806468
palmitate (16:0)	Long chain fatty acid	Lipid	0.228400124	-0.073504593
1-palmitoleoylglycerophosphocholine*	Lysolipid	Lipid	0.237496791	-0.067061977
glycochenodeoxycholate	Bile acid metabolism	Lipid	0.239276837	0.071154015
1-myristoylglycerophosphocholine	Lysolipid	Lipid	0.247535543	-0.065604981

1-stearoylglycerol (1-monostearin)	Monoacylglycerol	Lipid	0.249605311	0.068652254
arginine	Urea cycle; arginine-, proline-, metabolism	Amino acid	0.257809594	0.066241429
stearate (18:0)	Long chain fatty acid	Lipid	0.258101281	-0.068520439
1-palmitoylglycerophosphoethanolamine	Lysolipid	Lipid	0.259835857	0.063176676
2-stearoylglycerophosphocholine*	Lysolipid	Lipid	0.266476282	-0.062343175
succinylcarnitine	Krebs cycle	Energy	0.267035681	-0.074940177
isovalerate	Fatty acid metabolism	Lipid	0.269739105	0.0780315
tyrosine	Phenylalanine & tyrosine metabolism	Amino acid	0.280199458	-0.06536347
aspartate	Alanine and aspartate metabolism	Amino acid	0.294362477	-0.060150477
eicosenoate (20:1n9 or 11)	Long chain fatty acid	Lipid	0.295038148	-0.062732131
indoleacetate	Tryptophan metabolism	Amino acid	0.301003461	-0.058637472
citrulline	Urea cycle; arginine-, proline-, metabolism	Amino acid	0.302827972	-0.06241031
bilirubin (Z,Z)	Hemoglobin and porphyrin metabolism	Cofactors and vitamins	0.303434235	0.064667736
1-stearoylglycerophosphoethanolamine	Lysolipid	Lipid	0.30462107	0.059201259
theobromine	Xanthine metabolism	Xenobiotics	0.305595702	0.058838234
1-heptadecanoylglycerophosphocholine	Lysolipid	Lipid	0.309580515	-0.058127945
phosphate	Oxidative phosphorylation	Energy	0.311671029	-0.057351894
cortisol	Sterol/Steroid	Lipid	0.333152767	-0.059133617
cysteine	Cysteine, methionine, SAM, taurine metabolism	Amino acid	0.335529842	-0.054752263
glycine	Glycine, serine and threonine metabolism	Amino acid	0.339937937	0.054657052
2-oleoylglycerophosphocholine*	Lysolipid	Lipid	0.341152765	-0.053808122
taurochenodeoxycholate	Bile acid metabolism	Lipid	0.347432992	-0.063954724
alpha-hydroxyisovalerate	Valine, leucine and isoleucine metabolism	Amino acid	0.35064945	-0.054304998
gamma-glutamylglutamine	gamma-glutamyl	Peptide	0.350995997	0.057540149
threonate	Ascorbate and aldarate metabolism	Cofactors and vitamins	0.363096201	0.051878793
serotonin (5HT)	Tryptophan metabolism	Amino acid	0.366247529	-0.063937695

linoleate (18:2n6)	Essential fatty acid	Lipid	0.370272478	-0.052638849
bilirubin (E,E)*	Hemoglobin and porphyrin metabolism	Cofactors and vitamins	0.37777186	0.055143537
C-glycosyltryptophan*	Tryptophan metabolism	Amino acid	0.381847866	-0.056172654
1-palmitoylplasmenylethanolamine*	Lysolipid	Lipid	0.386280988	-0.05654387
glycerophosphorylcholine (GPC)	Glycerolipid metabolism	Lipid	0.386788825	-0.052969326
2-linoleoylglycerophosphocholine*	Lysolipid	Lipid	0.400935951	-0.053846124
glutamate	Glutamate metabolism	Amino acid	0.401448469	0.05234801
asparagine	Alanine and aspartate metabolism	Amino acid	0.402243558	0.049047728
gamma-glutamylphenylalanine	gamma-glutamyl	Peptide	0.40302834	-0.048761842
stearidonate (18:4n3)	Long chain fatty acid	Lipid	0.415863586	-0.046973876
decanoylcarnitine	Carnitine metabolism	Lipid	0.421308052	-0.048632379
phenylacetylglutamine	Phenylalanine & tyrosine metabolism	Amino acid	0.429323651	-0.044650467
pyroglutamine*	Glutamate metabolism	Amino acid	0.436302447	-0.047686836
isoleucine	Valine, leucine and isoleucine metabolism	Amino acid	0.442050346	0.045033858
2-tetradecenoyl carnitine	Carnitine metabolism	Lipid	0.443311091	-0.047970447
1,5-anhydroglucitol (1,5-AG)	Glycolysis, gluconeogenesis, pyruvate metabolism	Carbohydrate	0.447966612	-0.042238066
gamma-glutamylleucine	gamma-glutamyl	Peptide	0.454995113	0.048052456
butyrylcarnitine	Fatty acid metabolism (also BCAA metabolism)	Lipid	0.456535998	0.044891514
hippurate	Benzoate metabolism	Xenobiotics	0.464806863	-0.04160419
nonadecanoate (19:0)	Long chain fatty acid	Lipid	0.471975652	-0.042811212
lathosterol	Sterol/Steroid	Lipid	0.487762993	-0.046828288
myo-inositol	Inositol metabolism	Lipid	0.492979274	-0.039924102
2-hydroxybutyrate (AHB)	Cysteine, methionine, SAM, taurine metabolism	Amino acid	0.494636406	0.041355078
N-acetylthreonine	Glycine, serine and threonine metabolism	Amino acid	0.501013211	0.041642793
1-docosahexaenoylglycerophosphocholine*	Lysolipid	Lipid	0.504073288	0.038681534
erythritol	Sugar, sugar substitute, starch	Xenobiotics	0.504273249	-0.040920452
1-arachidonoylglycerophosphoinositol*	Lysolipid	Lipid	0.506008835	0.038832311

hypoxanthine	Purine metabolism, (hypo)xanthine/inosine containing	Nucleotide	0.506689948	-0.041504647
N-acetylornithine	Urea cycle; arginine-, proline-, metabolism	Amino acid	0.507989681	-0.039015485
proline	Urea cycle; arginine-, proline-, metabolism	Amino acid	0.510933148	0.038324022
pipecolate	Lysine metabolism	Amino acid	0.511214614	0.038712195
biliverdin	Hemoglobin and porphyrin metabolism	Cofactors and vitamins	0.513576937	-0.041996496
heptanoate (7:0)	Medium chain fatty acid	Lipid	0.515905704	-0.03750956
heme*	Hemoglobin and porphyrin	Cofactors and vitamins	0.516265494	0.040454297
1-arachidonoylglycerophosphoethanolamine*	Lysolipid	Lipid	0.517165282	0.034635495
acetylcarnitine	Carnitine metabolism	Lipid	0.526979852	-0.037742648
3-methylhistidine	Histidine metabolism	Amino acid	0.527075108	-0.039815141
fructose	Fructose, mannose, galactose, starch, and sucrose metabolism	Carbohydrate	0.536318919	-0.034709346
urate	Purine metabolism, urate metabolism	Nucleotide	0.538578514	-0.036892242
isovalerylcarnitine	Valine, leucine and isoleucine metabolism	Amino acid	0.540981941	0.036173178
phenyllactate (PLA)	Phenylalanine & tyrosine metabolism	Amino acid	0.546296164	0.040030297
1-eicosatrienoylglycerophosphocholine*	Lysolipid	Lipid	0.556717797	-0.031557187
creatine	Creatine metabolism	Amino acid	0.557417519	-0.034884732
threitol	Nucleotide sugars, pentose metabolism	Carbohydrate	0.567061379	0.036204913
10-undecenoate (11:1n1)	Medium chain fatty acid	Lipid	0.568501409	-0.033674985
dihomo-linolenate (20:3n3 or n6)	Essential fatty acid	Lipid	0.570249251	-0.032024724
piperine	Food component/Plant	Xenobiotics	0.57462635	-0.03454624
3-phenylpropionate (hydrocinnamate)	Phenylalanine & tyrosine metabolism	Amino acid	0.59532188	0.034557428
HWESASXX*	Polypeptide	Peptide	0.600257377	0.032659339

pantothenate	Pantothenate and CoA metabolism	Cofactors and vitamins	0.600786388	0.029913106
arabinose	Nucleotide sugars, pentose metabolism	Carbohydrate	0.604622176	-0.034496325
malate	Krebs cycle	Energy	0.605820052	-0.030793994
2-methylbutyroylcarnitine	Valine, leucine and isoleucine metabolism	Amino acid	0.609418527	0.03134199
trimethyl-N-aminovalerate	Carnitine metabolism	Lipid	0.611929477	0.030294397
2-aminobutyrate	Butanoate metabolism	Amino acid	0.616437861	-0.028989775
docosapentaenoate (n3 DPA; 22:5n3)	Essential fatty acid	Lipid	0.620637989	-0.029666529
erythrose	Fructose, mannose, galactose, starch, and sucrose metabolism	Carbohydrate	0.620704066	-0.028794522
gamma-glutamylvaline	gamma-glutamyl	Peptide	0.624233771	0.032031735
isobutyrylcarnitine	Valine, leucine and isoleucine metabolism	Amino acid	0.626874601	0.02850456
lysine	Lysine metabolism	Amino acid	0.629164721	0.027509215
uridine	Pyrimidine metabolism, uracil containing	Nucleotide	0.639425419	-0.025561525
ursodeoxycholate	Bile acid metabolism	Lipid	0.641617042	0.030680153
beta-hydroxyisovalerate	Valine, leucine and isoleucine metabolism	Amino acid	0.643680958	-0.026421585
p-cresol sulfate	Phenylalanine & tyrosine metabolism	Amino acid	0.648956732	0.027717284
quinate	Food component/Plant	Xenobiotics	0.651600708	0.028468533
leucine	Valine, leucine and isoleucine metabolism	Amino acid	0.661164279	0.02618625
stachydrine	Food component/Plant	Xenobiotics	0.66279498	0.024885199
citrate	Krebs cycle	Energy	0.665816808	-0.026428367
7-methylxanthine	Xanthine metabolism	Xenobiotics	0.667848407	-0.029461488
alpha-tocopherol	Tocopherol metabolism	Cofactors and vitamins	0.683161671	0.0264513
levulinate (4-oxovalerate)	Valine, leucine and isoleucine metabolism	Amino acid	0.689413123	0.023468372
cholesterol	Sterol/Steroid	Lipid	0.69119319	-0.024058929

stearoylcarnitine	Carnitine metabolism	Lipid	0.696627755	0.025051237
glycerol 2-phosphate	Chemical	Xenobiotics	0.705638198	0.025245225
1-oleoylglycerophosphoethanolamine	Lysolipid	Lipid	0.705806142	-0.020440552
5-oxoproline	Glutathione metabolism	Amino acid	0.707700086	-0.022046981
3-methyl-2-oxovalerate	Valine, leucine and isoleucine metabolism	Amino acid	0.708979647	0.023181994
propionylcarnitine	Fatty acid metabolism (also BCAA metabolism)	Lipid	0.713099385	-0.021460154
histidine	Histidine metabolism	Amino acid	0.715233191	-0.020942969
phenol sulfate	Phenylalanine & tyrosine metabolism	Amino acid	0.717169003	0.020114814
2-hydroxyisobutyrate	Valine, leucine and isoleucine metabolism	Amino acid	0.726024473	0.023359786
gamma-tocopherol	Tocopherol metabolism	Cofactors and vitamins	0.731312327	0.022187068
caproate (6:0)	Medium chain fatty acid	Lipid	0.733005356	0.019580302
indolepropionate	Tryptophan metabolism	Amino acid	0.736357193	-0.018992919
1-stearoylglycerophosphoinositol	Lysolipid	Lipid	0.73983159	-0.019447589
betaine	Glycine, serine and threonine metabolism	Amino acid	0.740691245	0.020232405
hexadecanedioate	Fatty acid, dicarboxylate	Lipid	0.76176838	0.01736582
allantoin	Purine metabolism, urate metabolism	Nucleotide	0.772327284	0.018445636
pyridoxate	Vitamin B6 metabolism	Cofactors and vitamins	0.773679178	0.016164233
docosahexaenoate (DHA; 22:6n3)	Essential fatty acid	Lipid	0.777859495	0.016840937
N-acetylalanine	Alanine and aspartate metabolism	Amino acid	0.782930259	-0.015871974
trans-4-hydroxyproline	Urea cycle; arginine-, proline-, metabolism	Amino acid	0.786623177	0.016193905
mannose	Fructose, mannose, galactose, starch, and sucrose metabolism	Carbohydrate	0.797845963	0.015033588
acetylphosphate	Oxidative phosphorylation	Energy	0.803652291	0.014422192
octadecanedioate	Fatty acid, dicarboxylate	Lipid	0.805675771	0.014785057
palmitoylcarnitine	Carnitine metabolism	Lipid	0.806553112	0.015236648

glycerate	Glycolysis, gluconeogenesis, pyruvate metabolism	Carbohydrate	0.807922561	0.013952507
kynurenine	Tryptophan metabolism	Amino acid	0.816852119	-0.013601649
valine	Valine, leucine and isoleucine metabolism	Amino acid	0.822365296	0.013067289
cholate	Bile acid metabolism	Lipid	0.823006759	0.014443729
cis-4-decenoyl carnitine	Carnitine metabolism	Lipid	0.83171795	-0.01489421
1-arachidonoylglycerophosphocholine*	Lysolipid	Lipid	0.851170359	-0.010214372
4-vinylphenol sulfate	Benzoate metabolism	Xenobiotics	0.854044156	0.011241448
erythronate*	Aminosugars metabolism	Carbohydrate	0.855544355	0.010574788
linolenate [alpha or gamma; (18:3n3 or 6)]	Essential fatty acid	Lipid	0.856771133	-0.01004328
taurolithocholate 3-sulfate	Bile acid metabolism	Lipid	0.860883795	0.009949856
3-methyl-2-oxobutyrate	Valine, leucine and isoleucine metabolism	Amino acid	0.861617288	0.011106901
scyllo-inositol	Inositol metabolism	Lipid	0.867239239	-0.011313734
glycocholate	Bile acid metabolism	Lipid	0.873174703	0.009899393
ornithine	Urea cycle; arginine-, proline-, metabolism	Amino acid	0.876325411	0.008774025
glycerol	Glycerolipid metabolism	Lipid	0.877508655	0.00945334
lactate	Glycolysis, gluconeogenesis, pyruvate metabolism	Carbohydrate	0.877583215	0.009231647
caprylate (8:0)	Medium chain fatty acid	Lipid	0.884490135	-0.009237127
tryptophan	Tryptophan metabolism	Amino acid	0.88903922	0.007214791
glutamine	Glutamate metabolism	Amino acid	0.894603784	-0.007886051
tetradecanedioate	Fatty acid, dicarboxylate	Lipid	0.895100641	-0.008360181
adrenate (22:4n6)	Long chain fatty acid	Lipid	0.895454801	0.007353593
urea	Urea cycle; arginine-, proline-, metabolism	Amino acid	0.895630464	-0.007780102
octanoylcarnitine	Carnitine metabolism	Lipid	0.896083838	-0.007645623
catechol sulfate	Benzoate metabolism	Xenobiotics	0.899970581	0.007115516
dodecanedioate	Fatty acid, dicarboxylate	Lipid	0.902395873	0.007199001
1-palmitoylglycerophosphoinositol*	Lysolipid	Lipid	0.907065383	-0.008186688
3-hydroxybutyrate (BHBA)	Ketone bodies	Lipid	0.909389092	-0.006835149
pelargonate (9:0)	Medium chain fatty acid	Lipid	0.921051245	-0.006192266

tryptophan betaine	Tryptophan metabolism	Amino acid	0.921975247	0.006740114
4-methyl-2-oxopentanoate	Valine, leucine and isoleucine metabolism	Amino acid	0.92230276	-0.006119761
glycerol 3-phosphate (G3P)	Glycerolipid metabolism	Lipid	0.923359305	0.006141396
carnitine	Carnitine metabolism	Lipid	0.923569427	0.005721227
4-ethylphenylsulfate	Benzoate metabolism	Xenobiotics	0.923754881	-0.006568308
1-linoleoylglycerophosphoethanolamine*	Lysolipid	Lipid	0.926538171	-0.005371443
2-hydroxystearate	Fatty acid, monohydroxy	Lipid	0.932667255	-0.004356958
N-acetylglycine	Glycine, serine and threonine metabolism	Amino acid	0.932743716	-0.006084948
1-palmitoylglycerol (1-monopalmitin)	Monoacylglycerol	Lipid	0.933762073	-0.004766886
7-alpha-hydroxy-3-oxo-4-cholestenoate (7-Hoca)	Sterol/Steroid	Lipid	0.933879719	-0.005018504
eicosapentaenoate (EPA; 20:5n3)	Essential fatty acid	Lipid	0.935924715	0.00487171
3-carboxy-4-methyl-5-propyl-2-furanpropanoate (CMPF)	Fatty acid, dicarboxylate	Lipid	0.953413774	0.003645768
phenylalanine	Phenylalanine & tyrosine metabolism	Amino acid	0.957426884	-0.002758028
hexanoylcarnitine	Carnitine metabolism	Lipid	0.958405703	0.003261256
pro-hydroxy-pro	Dipeptide	Peptide	0.968192839	0.001555603
benzoate	Benzoate metabolism	Xenobiotics	0.982064166	0.001150327
glucose	Glycolysis, gluconeogenesis, pyruvate metabolism	Carbohydrate	0.986789188	0.001080741
3-indoxyl sulfate	Tryptophan metabolism	Amino acid	0.989460695	-0.00070736
oleoylcarnitine	Carnitine metabolism	Lipid	1	0.000161859
